# Author Index

**DOI:** 10.1111/jdi.12671

**Published:** 2017-05-05

**A**

Abe, M., AII‐P‐15, AI‐P‐21, AII‐P‐26

Aburatani, H., AII‐6‐8

Ahn, C., AI‐P‐32, AI‐P‐43

Ahn, KJ., AII‐P‐31

Aihara, H., AII‐6‐15

Akagawa, H., AII‐P‐40

Akasaka, H., AI‐P‐39

Akesaka, K., AII‐P‐22

Akuzawa, M., AII‐P‐46

Alejandria, M., AI‐6‐15

An, K., AII‐5‐1

Andou, K., AII‐P‐46

Ang, LC., AI‐P‐16

Arimura, E., AII‐P‐15

Atsumi, T., AII‐P‐21

Azuma, Y., AI‐P‐31

**B**

Baba, M., AI‐P‐37

Bae, JC., AI‐P‐7

Baek, HS., AI‐P‐19

Baik, SH., AII‐P‐31

Bao, Y., AII‐P‐44, AI‐6‐13, AI‐6‐8, AI‐P‐20, AI‐P‐34, AI‐P‐44, AI‐P‐5, AI‐P‐8

Bau, C‐T., AI‐P‐47

Bavuu, O., AI‐P‐40

Bee, YM., AI‐P‐16

Bernardo, DC., AI‐P‐38

Bhonde, R., AII‐P‐5

Boldbaatar, T., AI‐P‐40

Bolze, S., AI‐6‐6, AII‐6‐13

Byambatsogt, B., AI‐P‐22

**C**

Cai, X., AI‐P‐10, AI‐6‐4

Carmean M., C., AII‐P‐4

Ceralde, A., AII‐P‐39

Cha, B., AII‐P‐29

Cha, BY., AI‐6‐9

Chan, ETC., AI‐P‐16

Chandravanshi, B., AII‐P‐5

Chang, T‐J., AII‐P‐36

Chen Hung, C., AI‐P‐45

Chen, C‐H., AI‐P‐42

Chen, CS., AI‐P‐26

Chen, C‐T., AI‐5‐3

Chen, C‐Y., AI‐5‐3

Chen, H‐J., AII‐P‐36

Chen, P., AI‐6‐13

Chen, S., AII‐6‐11, AI‐6‐13

Chen, T‐L., AI‐P‐28, AII‐5‐4

Chen, W., AII‐P‐16

Chen, Y., AI‐P‐5, AI‐P‐44, AI‐6‐4

Chen, Y‐H., AII‐P‐36

Chen, Y‐L., AI‐P‐30

Cheng, P‐S., AI‐P‐26

Cheung, LTF., AII‐P‐48

Cho H, N., AII‐6‐9

Cho, JH., AI‐6‐9

Cho, KY., AII‐P‐21

Cho, SK., AII‐P‐31

Choi, GKT., AII‐P‐48

Choi, HS., AII‐6‐9

Choi, S., AI‐6‐1

Choi, Y‐H., AII‐5‐2, AII‐5‐3

Chon, S., AII‐P‐31

Chou, Y‐C., AII‐5‐4

Chuang, L‐M., AII‐P‐36

Chukwuma, CI., AII‐P‐17

Chung, FCC., AII‐P‐48

Chung, Y‐W., AI‐P‐42

**D**

Dauchy, S., AI‐6‐6

Deng, Y., AII‐6‐11

Dominguez, AM., AII‐P‐39

**E**

Ebisui, O., AII‐P‐22

**F**

Faltado Lumbera, A. Jr, AII‐P‐39

Fang, Q., AII‐6‐11

Fauzi, M., AII‐P‐7

Fernando, PHP., AI‐P‐14

Fouqueray, P., AI‐6‐6, AII‐6‐13

Francisco, C., AI‐6‐15

Fujikawa, J., AII‐P‐26

Fujimoto, H., AI‐5‐1

Fujita, N., AI‐5‐1

Fujiwara, T., AI‐P‐39

**G**

Gao, P., AII‐P‐20

Gao, X., AI‐6‐4, AI‐P‐10

Ge, X., AI‐P‐44, AI‐P‐5

Geng, Y., AI‐6‐2

Goh, S‐Y., AI‐P‐16

Gonzales, E., AI‐6‐15

Guo, J., AI‐P‐27

Gushiken, M., AII‐P‐32

**H**

Ha, IG., AII‐P‐31

Ha, KH., AII‐5‐5

Hallakou‐Bozec, S., AII‐6‐13

Hamada, Y., AII‐P‐30

Hamamatsu, K., AI‐5‐1

Hamamoto, Y., AI‐6‐3, AI‐6‐5, AI‐P‐29, AII‐P‐23, AII‐P‐24, AII‐P‐26, AII‐P‐35

Hamasaki, A., AI‐P‐21, AII‐P‐26

Han, C‐J., AII‐5‐3

Han, J., AII‐6‐11

Han, K., AII‐5‐1

Han, SW., AII‐P‐31

Han, X., AI‐P‐10, AI‐P‐2

Hara, K., AII‐6‐8

Hara, Y., AII‐P‐22

Haraguchi, T., AI‐6‐5

Harashima, S‐i., AII‐P‐13, AII‐P‐27, AII‐P‐9

Hatakeyama, S., AII‐P‐1

He, R., AI‐P‐36

He, X., AI‐P‐20

He, Z., AII‐P‐12

Herrera L, P., AI‐5‐5

Higashiyama, H., AII‐P‐35

Himathongkam, T., AII‐P‐28

Hirayama, K., AII‐P‐38

Hodai, Y., AI‐P‐31

Hong, E., AI‐6‐1

Hong, J., AI‐P‐9

Hong, W‐J., AI‐P‐7

Honjo, S., AI‐P‐21, AII‐P‐26

Horiguchi, K., AII‐6‐12, AII‐P‐11

Horiuchi, M., AII‐P‐15

Hoshikawa, R., AII‐P‐4

Hotta, R., AI‐P‐37

Hou, W., AII‐P‐45

Hou, X., AI‐6‐13, AII‐P‐49

Hu, C., AII‐P‐49, AII‐P‐6, AI‐5‐6, AI‐P‐1, AI‐P‐12, AI‐P‐25, AI‐P‐3, AI‐P‐4, AI‐P‐46, AI‐P‐6, AII‐6‐7, AII‐P‐12, AII‐P‐14, AII‐P‐47

Hu, Q‐S., AII‐P‐19

Hu, X., AI‐P‐34, AI‐P‐8

Huai‐Ching, I‐TL., AII‐P‐34

Huang, S‐F., AII‐P‐36

Huang, ST., AI‐P‐42

Huang, Y., AII‐6‐7

Hur, KY., AI‐P‐7

Hwang, YC., AI‐P‐7

Hyo, T., AII‐P‐24, AI‐6‐3, AI‐P‐29

**I**

Ikeda, K., AII‐P‐34

Ikeda, Y., AII‐6‐15

Ikema, T., AI‐6‐10

Ikematsu, T., AI‐6‐10

Inagaki, A., AI‐P‐31

Inagaki, N., AI‐5‐1, AII‐P‐13, AII‐P‐27, AII‐P‐34, AII‐P‐7, AII‐P‐9

Inoue, H., AI‐P‐13

IshII, S., AI‐P‐15, AII‐6‐12, AII‐P‐11

Ishikawa, K., AI‐P‐13

Ishimura, N., AI‐P‐18

Islam, MS., AII‐P‐17

Ito, T., AI‐P‐33

Itoh, Y., AI‐6‐6

Iwakura, T., AII‐P‐24

Iwane, A., AII‐6‐8

Iwasaki, M., AI‐6‐5

Iwasaki, N., AII‐P‐40

**J**

Jang, SS., AI‐P‐45

Jeon, JY., AII‐5‐5

Jeon, YK., AI‐6‐14

Ji, L., AI‐6‐4, AI‐P‐10

Ji, l., AI‐P‐2

Jia, W., AI‐P‐44, AI‐5‐6, AI‐6‐13, AI‐6‐8, AI‐P‐1, AI‐P‐12, AI‐P‐20, AI‐P‐25, AI‐P‐3, AI‐P‐34, AI‐P‐36, AI‐P‐4, AI‐P‐46, AI‐P‐5, AI‐P‐6, AI‐P‐8, AII‐6‐11, AII‐6‐7, AII‐P‐10, AII‐P‐44, AII‐P‐47, AII‐P‐49, AII‐P‐6

Jia, W‐P., AII‐P‐14

Jiang, F., AI‐P‐3, AI‐5‐6, AI‐P‐1

Jiang, Y‐D., AII‐P‐36

Jin, HY., AI‐P‐19

Jin, L., AI‐P‐46

Jin, M., AII‐6‐15

Jin, S‐M., AI‐P‐7

Jirarungsaritkul, S., AII‐P‐28

Joo, E., AII‐P‐34

Joung, KH., AII‐P‐18

Ju, L., AII‐6‐11

Juang, J‐H., AI‐5‐3

**K**

Kadowaki, T., AII‐6‐8

Kanada, S., AI‐6‐5

Kanamori, A., AII‐P‐24

Kang, B., AII‐5‐2

Kang, E., AII‐P‐29

Kang, YH., AI‐P‐24

Kao, C‐D., AI‐P‐47

Kao, C‐W., AI‐5‐3

Kashima, R., AII‐P‐27

Katano, AT., AII‐6‐12

Katano‐Toki, A., AII‐P‐11, AII‐P‐46

Kato, M., AI‐P‐41

Kato, N., AI‐P‐41

Kato, T., AI‐P‐31

Kawaguchi, H., AII‐P‐15

Kawahara, N., AI‐P‐39

Kergoat, M., AII‐6‐13

Khang, AR., AI‐P‐24

Khattri, S., AI‐6‐11

Kim, BH., AI‐6‐14

Kim, DJ., AII‐5‐5

Kim, D‐J., AI‐P‐9

Kim, D‐M., AII‐P‐25

Kim, E., AI‐6‐14

Kim, HJ., AII‐5‐5, AII‐P‐18

Kim, H‐S., AI‐6‐9

Kim, I., AI‐6‐14

Kim, J., AI‐5‐4, AI‐P‐43, AI‐P‐32

Kim, JH., AI‐P‐7, AI‐6‐14

Kim, JT., AII‐6‐9, AII‐P‐18

Kim, J‐W., AII‐P‐2, AII‐P‐3

Kim, K., AI‐P‐32, AI‐P‐43

Kim, KJ., AI‐6‐12

Kim, KS., AII‐P‐18

Kim, MJ., AII‐P‐2

Kim, S., AII‐P‐29

Kim, SH., AII‐P‐31

Kim, S‐W., AI‐P‐11

Kim, Y., AI‐P‐32, AI‐P‐43

Kimura, H., AI‐5‐1

Kinoshita, T., AI‐P‐31

Kira, D., AII‐P‐38

Kirkley G., A., AII‐P‐4

Kishida, S., AII‐P‐37

Kishimoto, M., AI‐P‐18

Kitamura, Y., AII‐P‐40

Kitano, N., AII‐P‐24

Kitatani, N., AI‐6‐3

Ko, R., AII‐P‐27

Ko, S‐Y., AII‐5‐3

Kobayashi, A., AI‐P‐13

Kobayashi, I., AII‐P‐46

Kobayashi, M., AII‐6‐8

Kobayashi, N., AII‐6‐8

Koduka, C., AI‐6‐10

Koh, G., AI‐6‐7

Koike, M., AI‐P‐33

Kondo, Y., AI‐P‐33

Koshian, F., AI‐6‐3

Koshima, I., AII‐6‐8

Kouchi, Y., AII‐P‐38

Krittiyawong, S., AII‐P‐28

Ku, BJ., AII‐P‐18

Kubota, A., AII‐P‐24

Kubota, M., AI‐P‐39

Kubota, N., AII‐6‐8

Kubota, S., AI‐P‐29

Kumagai, J., AI‐P‐13

Kumar, MS., AI‐6‐11

Kumar, PS., AI‐6‐11

Kuo, C‐H., AI‐5‐3

Kuramoto, H., AII‐P‐38

Kurimoto, J., AI‐P‐31

Kurita, K., AI‐P‐13

Kuroe, A., AII‐P‐24

Kurose, T., AI‐6‐3, AI‐6‐5, AI‐P‐29, AII‐P‐23, AII‐P‐24, AII‐P‐35

Kushiro, R., AII‐P‐38

Kuwamura, Y., AII‐P‐37, AI‐P‐39

Kuwata, H., AI‐6‐3, AI‐6‐5, AI‐P‐29, AII‐P‐24

Kwon, H., AII‐5‐1

**L**

Lai, Y‐C., AII‐P‐36

Lat, AM., AI‐P‐38

Lee, BW., AII‐P‐29

Lee, C‐L., AI‐P‐42

Lee, EY., AI‐6‐9

Lee, E‐Y., AII‐5‐2

Lee, HK., AII‐6‐9

Lee, HW., AI‐P‐24

Lee, I‐K., AI‐P‐11

Lee, J., AII‐5‐1

Lee, JH., AI‐6‐12, AII‐P‐18

Lee, J‐H., AII‐5‐2, AII‐5‐3

Lee, KA., AI‐P‐19

Lee, KW., AII‐P‐31

Lee, K‐W., AII‐5‐5

Lee, M., AII‐P‐25

Lee, MJ., AI‐6‐14

Lee, M‐K., AI‐P‐7

Lee, S., AI‐P‐32, AI‐P‐43

Lee, SA., AI‐6‐7

Lee, SH., AII‐P‐3

Lee, S‐H., AI‐6‐9

Lee, Y., AII‐P‐29

Leung, PS., AII‐P‐8

Li, M., AI‐P‐44, AI‐P‐5

Li, X., AII‐P‐10, AI‐P‐27

Li, Y., AI‐P‐6, AI‐P‐2

Liao, C‐C., AI‐P‐28, AII‐5‐4

Lim, S‐Y., AII‐5‐2, AII‐5‐3

Lin, J‐L., AI‐P‐47

Liu, F., AII‐P‐45, AI‐P‐36

Liu, J., AII‐P‐20

Liu, L., AI‐P‐5, AI‐P‐44

Liu, Y., AII‐P‐13, AII‐P‐9, AII‐P‐49

Liu, Z‐C., AII‐P‐19

Lo, Y‐C., AI‐P‐42

Luo, D., AI‐5‐2

Luo, Y., AI‐6‐8, AI‐P‐34, AI‐P‐8, AII‐P‐44

Lv, X., AI‐6‐2

**M**

Ma, X., AI‐P‐8, AI‐6‐8, AI‐P‐20, AI‐P‐34, AII‐P‐44

Ma, Y., AI‐P‐2

Macalalad‐Josue, AA., AII‐5‐6

Maeda, Y., AI‐P‐17

Maejima, Y., AI‐5‐5

Makabe, N., AI‐6‐3

Maningat, P., AI‐6‐15

Maningat, PD., AII‐P‐39

Mano, F., AII‐P‐34

Marushita, R., AI‐P‐33

Masuzaki, H., AI‐6‐10

Matsuhisa, M., AII‐P‐37

Matsumoto, S., AII‐6‐12, AII‐P‐11

McMorran, D., AI‐P‐37

Meng, S., AI‐6‐2

Meng, X., AII‐P‐45

Mim, JM., AII‐P‐18

Mimura, M., AI‐P‐17

Min, K., AII‐5‐1

Mina, A., AI‐6‐2

Minami, K., AI‐5‐5

Miyamoto, L., AII‐6‐15

Miyoshi, H., AII‐P‐21

Mizokami, T., AII‐P‐1

Monma, N., AI‐P‐33

Mopuri, R., AII‐P‐17

Mori, M., AII‐6‐12, AII‐P‐11

Moriwaki, R., AI‐P‐17

Munkhtur, B., AII‐P‐33

Murakami, T., AI‐5‐1, AII‐P‐27

Murayama, Y., AI‐6‐10

**N**

Nagai, Y., AI‐P‐18

Nagashima, K., AII‐P‐27, AII‐P‐7

Nagata, T., AII‐P‐37

Nakajima, Y., AII‐P‐46, AI‐P‐23, AII‐6‐12, AII‐P‐11

Nakamura, A., AII‐P‐21, AII‐P‐38

Nakandakari, M., AI‐6‐10

Nakasatien, S., AII‐P‐28

Nam, J., AI‐P‐32, AI‐P‐43

Nam, MS., AII‐P‐31

Nata, K., AII‐P‐1

Nemoto, K., AI‐P‐17

Nishikawa, T., AI‐P‐17

Niu, X., AI‐5‐2

Noh, J., AI‐P‐9

Noh, Y., AI‐6‐1

Nonogaki, K., AII‐6‐14, AI‐P‐35

Nukada, H., AI‐P‐37

Nukuda, M., AI‐P‐33

**O**

Oduori S, O., AI‐5‐5

Ogasawara, S., AI‐P‐37

Ogata, M., AII‐P‐40

Ogura, M., AII‐P‐27, AII‐P‐7

Oh, MS., AII‐6‐9

Oh, SY., AI‐6‐14

Ohno, K., AII‐P‐22

Oida, K., AII‐P‐24

Okada, S., AII‐P‐42, AI‐P‐15, AI‐P‐23, AII‐6‐10, AII‐P‐11

Okamoto, S., AI‐P‐29

Okamura, E., AII‐P‐26, AI‐P‐21

Okamura, K., AI‐P‐29

Okamura, T., AII‐6‐12

Okazaki, Y., AII‐6‐8

Okura, M., AII‐P‐34

Omlor, Y., AII‐P‐32

Omura, M., AI‐P‐33

Osaki, A., AI‐P‐23, AII‐6‐10

Ota, K., AI‐P‐33

Ozawa, A., AI‐P‐15, AII‐6‐12, AII‐P‐11

**P**

Pacaira, JPP., AII‐P‐41

Pak Kim, Y., AII‐6‐9

Pan, J., AII‐P‐45

Pan, X., AI‐P‐34, AI‐P‐8, AII‐P‐44

Park, J., AI‐P‐32, AI‐P‐43, AII‐P‐25

Park, K., AI‐P‐32, AI‐P‐43

Park, S., AII‐P‐25

Park, TS., AI‐P‐19

Park, WH., AII‐6‐9

Park, Y., AII‐P‐31

Peng, H‐Y., AII‐P‐36

Puchi, B., AI‐6‐2

**R**

Reganit, PF., AI‐6‐15

Ren, Q., AI‐6‐2

Ren, W., AI‐P‐27

Rhee, SY., AII‐P‐31

**S**

Sagawa, M., AI‐P‐33

Saito, K., AII‐P‐40

Saito, S., AI‐P‐39

Saito, T., AI‐P‐23, AII‐6‐10

Saji, H., AI‐5‐1

Sakai, S., AII‐P‐38

Sakamaki, K., AII‐P‐46

Sakamoto, E., AII‐P‐37

Sakiyama, Y., AII‐P‐38

Sakuramachi, Y., AII‐P‐23, AI‐P‐29

Salvana, EM., AI‐6‐15

Sanjeewani, AN., AI‐P‐14

Sarakom, S., AII‐P‐28

Sargis M, R., AII‐P‐4

Sarmiento, AJ., AII‐P‐43

Sasako, T., AII‐6‐8

Sato, H., AI‐P‐33

Sato, T., AI‐P‐31, AI‐P‐23, AII‐6‐10

Satoh, T., AI‐P‐15, AII‐6‐12, AII‐P‐11, AII‐P‐46

Seino, S., AI‐5‐5, AII‐P‐4

Seino, Y., AI‐6‐3, AI‐6‐5, AI‐P‐29, AII‐P‐23, AII‐P‐24, AII‐P‐35

Shan, AKP., AII‐P‐48

Sheen, Y‐J., AI‐P‐47

Shen, Y., AI‐6‐8

Shen, Z., AII‐P‐16

Sheu, WH‐H., AI‐P‐47

Shibusawa, N., AI‐P‐15, AII‐6‐12, AII‐P‐11

Shibusawa, R., AI‐P‐23, AII‐6‐10

Shimabukuro, M., AI‐6‐10

Shimoda, Y., AI‐P‐23, AII‐6‐10

Shimomura, K., AI‐5‐5

Shimomura, Y., AII‐P‐46

Shong, M., AII‐P‐18

Shu, K‐H., AI‐P‐42

Singh, SY., AI‐6‐11

Sirakura, T., AI‐6‐10

Sison, O., AI‐6‐15

Sison‐Pena, CM., AI‐P‐38

Son, SM., AI‐P‐24

Song, J., AI‐6‐2

Song, Y., AII‐P‐19

Sonomtseren, S., AI‐P‐22, AI‐P‐40

Su, H., AI‐P‐20

Sugawara, K., AII‐P‐4

Suh, B‐K., AII‐5‐2

Sumikawa, M., AI‐P‐39, AII‐P‐37

Sumikawa, Y., AI‐P‐39

Sumita, K., AI‐6‐5

Sun, Y., AI‐5‐2

Sunagawa, S., AI‐6‐10

Suwanai, H., AII‐6‐8

Suzuki, H., AII‐P‐24

**T**

Tagaya, Y., AI‐P‐23, AII‐6‐10

Tahara, Y., AII‐P‐7

Tajima, N., AI‐P‐18

Takagi, Y., AII‐P‐38

Takahashi, H., AI‐5‐5

Takahashi, I., AII‐P‐1

Takahashi, N., AII‐P‐24

Takamizawa, T., AII‐6‐12

Takayanagi, Y., AI‐P‐17

Takayasu, H., AI‐P‐33

Takeda, K., AII‐6‐13

Takenaka, T., AII‐P‐38

Takikawa, I., AII‐P‐37

Tamaki, M., AII‐P‐22

Tamaki, T., AII‐6‐15

Tamura, M., AI‐6‐10

Tan, AXH., AI‐P‐16

Tanaka, N., AI‐6‐3, AI‐P‐29, AII‐P‐24, AII‐P‐35, AII‐6‐15

Tanaka, R., AI‐P‐33

Tang, C., AI‐6‐6

Tatewaki, K., AI‐P‐39

Tatsuoka, H., AII‐P‐7

Teh, MM., AI‐P‐16

Thewjitcharoen, Y., AII‐P‐28

Tien, K‐J., AI‐P‐30

Tokumoto, S., AI‐P‐21, AII‐P‐7

Tokunaga, H., AII‐P‐22

Tomaru, T., AI‐P‐15, AII‐6‐12, AII‐P‐11

Tomida, Y., AII‐6‐15

Toshima, H., AI‐P‐41

Toyoda, K., AI‐5‐1

Tsai, S‐F., AI‐P‐42

Tserensodnom, S., AI‐P‐22

Tsuchiya, E., AI‐P‐17

Tsuchiya, K., AII‐6‐15

**U**

Uchida, Y., AII‐P‐34

Uchigata, Y., AII‐P‐40

Ueda, T., AII‐P‐22

Ueki, K., AII‐6‐8

Uemura, H., AII‐P‐37

Ueno, S., AII‐P‐35, AI‐P‐29

Ushikai, M., AII‐P‐15

Usman, K., AI‐6‐11

Usui, R., AII‐P‐7, AII‐P‐9

**W**

Wada, E., AI‐P‐31

Wada, Y., AII‐P‐26

Wakutsu, M., AI‐P‐33

Wang, C., AI‐5‐2, AII‐P‐10

Wang, C‐S., AII‐P‐36

Wang, J., AII‐P‐14

Wang, S., AII‐P‐10, AI‐P‐1

Wang, T., AI‐P‐12, AI‐P‐36

Wang, W., AII‐P‐49

Wang, X., AI‐P‐25

Wang, Y., AI‐5‐2, AII‐P‐8, AII‐P‐9, AII‐P‐13, AI‐P‐20

Wang, Y‐J., AII‐P‐36

Wanothayaroj, E., AII‐P‐28

Watanabe, K., AI‐6‐3, AI‐P‐29, AII‐P‐24

Watanabe, T., AI‐P‐15, AII‐6‐12

Wee, Z., AI‐P‐16

Wei, P., AII‐P‐14

Wei, PJ., AII‐P‐12

Wickramarathne, DBM., AI‐P‐14

Wijang, PP., AII‐P‐15

Woo, JT., AII‐P‐31

Wu, C‐Y., AI‐P‐42

Wu, J., AI‐6‐13

Wu, M‐J., AI‐P‐42

**X**

Xi, G., AI‐P‐27

Xiao, Y., AI‐6‐8, AII‐P‐44

Xin, X., AI‐P‐16

Xiong, Q., AI‐6‐8, AI‐P‐34, AI‐P‐8, AII‐P‐44

Xu, B., AI‐P‐4

Xu, G., AII‐P‐16

Xu, W., AII‐6‐15

Xu, Y., AI‐6‐8, AI‐P‐34, AI‐P‐8, AII‐P‐44

**Y**

Yabe, D., AI‐6‐5, AII‐P‐23, AII‐P‐27, AII‐P‐7

Yagihashi, S., AI‐P‐37

Yamada, E., AII‐6‐10, AI‐P‐23

Yamada, M., AI‐P‐15, AI‐P‐23, AII‐6‐10, AII‐6‐12, AII‐P‐11, AII‐P‐42, AII‐P‐46

Yamaguchi, K., AI‐P‐31

Yamamoto, J., AI‐P‐33

Yamamoto, K., AII‐P‐21

Yamaoka, T., AII‐6‐15

Yamato, H., AII‐P‐37

Yamauchi, T., AII‐6‐8

Yamazaki, S., AI‐P‐33

Yamazato, H., AI‐6‐5

Yan, D., AII‐P‐47

Yan, F., AII‐P‐49

Yan, J., AI‐5‐6

Yan, J., AII‐6‐7

Yanase, K., AII‐P‐1

Yang, E‐J., AI‐6‐7

Yang, J., AII‐6‐11

Yang, L., AI‐6‐2

Yang, S., AI‐6‐2

Yang, W., AI‐6‐4

Yang, Y., AI‐6‐9, AII‐5‐2, AII‐6‐11

Yanjmaa, E., AII‐P‐33

Yasuda, K., AII‐P‐24

Yenseung, N., AII‐P‐28

Yi, DW., AI‐P‐24

Yi, H‐S., AII‐P‐18

Yin, J., AI‐P‐5, AI‐P‐44, AI‐P‐20

Yokoh, H., AI‐P‐13

Yokoi, N., AI‐5‐5, AII‐P‐4

Yokote, K., AI‐P‐13

Yokoyama, H., AII‐P‐24

Yoo, H‐J., AII‐5‐3

Yoo, S., AI‐6‐7

Yoon, K., AII‐P‐25

Yoon, K‐H., AI‐5‐4, AI‐6‐9, AII‐5‐2, AII‐5‐3, AII‐P‐2, AII‐P‐3

Yoshiji, S., AII‐P‐27

Yoshimura, K., AII‐6‐8

Yoshino, S., AII‐P‐11, AII‐6‐12

You, J., AI‐P‐32

You, Y‐H., AII‐P‐2

You, Y‐HE., AI‐5‐4

Yu, D‐M., AI‐P‐42

Yu, J., AI‐P‐43

Yu, MG., AI‐6‐15

**Z**

Zhang, J., AI‐P‐5, AI‐P‐44

Zhang, R., AI‐P‐1, AI‐P‐12, AI‐P‐25, AI‐P‐44, AI‐P‐5, AII‐P‐12

Zhang, X., AII‐6‐11, AI‐6‐8

Zhao, A., AII‐P‐45

Zhao, T., AI‐5‐2

Zhao, W., AI‐P‐44, AI‐P‐5, AII‐P‐45

Zhao, Z‐y., AII‐P‐19

Zhou, J., AI‐P‐20

Zhou, L., AI‐6‐2

Zhou, X., AI‐P‐2

Zhu, H., AII‐P‐10, AI‐5‐2

Zhuang, L., AI‐P‐44, AI‐P‐5

Zong, W., AII‐P‐49

